# STAP-2 facilitates insulin signaling through binding to CAP/c-Cbl and regulates adipocyte differentiation

**DOI:** 10.1038/s41598-024-56533-0

**Published:** 2024-03-09

**Authors:** Yuichi Sekine, Kazuna Kikkawa, Sachie Honda, Yuto Sasaki, Shoya Kawahara, Akihiro Mizushima, Sumihito Togi, Masahiro Fujimuro, Kenji Oritani, Tadashi Matsuda

**Affiliations:** 1 Department of Cell Biology, Kyoto Pharmaceutical University, Kyoto, 607-8412 Japan; 2 Department of Immunology, Graduate School of Pharmaceutical Sciences, Hokkaido University, Sapporo, 060-0812 Japan; 3 Division of Genomic Medicine, Department of Advanced Medicine, Medical Research Institute, Kanazawa Medical University, Kahoku, Ishikawa 920-0293 Japan; 4 Department of Hematology, International University of Health and Welfare, Narita, Chiba 286-8686 Japan

**Keywords:** Cell signalling, Insulin signalling

## Abstract

Signal-transducing adaptor protein-2 (STAP-2) is an adaptor molecule involved in several cellular signaling cascades. Here, we attempted to identify novel STAP-2 interacting molecules, and identified c-Cbl associated protein (CAP) as a binding protein through the C-terminal proline-rich region of STAP-2. Expression of STAP-2 increased the interaction between CAP and c-Cbl, suggesting that STAP-2 bridges these proteins and enhances complex formation. CAP/c-Cbl complex is known to regulate GLUT4 translocation in insulin signaling. STAP-2 overexpressed human hepatocyte Hep3B cells showed enhanced GLUT4 translocation after insulin treatment. Elevated levels of *Stap2* mRNA have been observed in 3T3-L1 cells and mouse embryonic fibroblasts (MEFs) during adipocyte differentiation. The differentiation of 3T3-L1 cells into adipocytes was highly promoted by retroviral overexpression of STAP-2. In contrast, STAP-2 knockout (KO) MEFs exhibited suppressed adipogenesis. The increase in body weight with high-fat diet feeding was significantly decreased in STAP-2 KO mice compared to WT animals. These data suggest that the expression of STAP-2 correlates with adipogenesis. Thus, STAP-2 is a novel regulatory molecule that controls insulin signal transduction by forming a c-Cbl/STAP-2/CAP ternary complex.

## Introduction

Signal-transducing adaptor protein-2 (STAP-2) is an adaptor protein which carries a PH domain, a SH2-like domain, and a proline-rich region^1,2^. STAP-2 modulates intracellular signaling pathways in response to various cytokines and growth factors by interacting with other proteins through its domain structure. Cytokine-induced Jak/STAT signaling^1,3,4^, macrophage-colony stimulating factors-induced PI3k/Akt activation^5,6^, and epidermal growth factor (EGF)-stimulated receptor signaling^7^ are finely regulated by STAP-2. The expression of STAP-2 is ubiquitous in human and mouse tissues^8^ and is also strongly detected in some tumor cells, such as breast tumor cell lines T47D and MCF7^8^, and a prostate cancer cell line DU145^7^. Notably, STAP-2 is inducible in some tissues and cell lines in response to several stimuli. *Stap2* mRNA is strongly elevated in mouse livers after injection of lipopolysaccharide (LPS). In addition, *Stap2* mRNA expression is upregulated in M1 cells, a murine myeloid leukemia cell line, after stimulation with the IL-6 family cytokine leukemia inhibitory factor^1^. Tyrosine residues in STAP-2 are phosphorylated by several kinases, and this phosphorylation is vital for regulating signal transduction^1,4,7,9^. As STAP-2 has no enzymatic or effector functions, its function as an adaptor protein is thought to be regulated by its mRNA induction and phosphorylation activities.

Using a yeast two-hybrid system screen of a mouse embryo cDNA library with human STAP-2 (amino acids 1–403) as bait^9^, we identified c-Cbl associated protein (CAP) as a new STAP-2-binding partner. Our previous studies have reported that STAP-2 regulates functions of key molecules in various signaling systems through the binding; therefore, STAP-2 may possibly modify CAP functions. CAP is an adaptor protein carrying a SH3 domain^10^. It is expressed a variety of tissues and cells, such as skeletal muscle and adipose tissues. CAP functions to regulate cell adhesion and migration, and membrane trafficking, as well as intracellular signaling^11^. In addition, CAP regulates the insulin-dependent tyrosine phosphorylation of c-Cbl^12^. c-Cbl/CAP interactions are involved in glucose transporter type 4 (GLUT4) trafficking from the cytoplasm to the plasma membrane, resulting in increased glucose uptake^13–15^.

Adipogenesis is a tightly controlled cellular differentiation process, in which mesenchymal stem cells commit to preadipocytes followed by differentiating into adipocytes. These differentiation steps are mainly controlled by peroxisome proliferator-activated receptor γ (PPARγ) and CCAAT enhancer-binding proteins (C/EBPs). Insulin regulates anabolic responses in adipose tissues through promoting glucose and free fatty acid uptake and through inhibiting lipolysis(ref)^16^. In addition, insulin controls proliferation and differentiation of adipocytes through promoting the gene expression of several fat-specific transcription factors, such as SREBP-1c and PPARγ^17,18^.

In this study, we focus on effects of STAP-2 on adipocytes whose differentiation steps are dependent on CAP/insulin-signaling. We here demonstrated STAP-2 expression enhances complex formation of CAP and c-Cbl to increase signals from insulin/insulin receptor systems. STAP-2 deficient mice show diminished increase of body size and liver weight after high-fat diet condition. Therefore, STAP-2 plays a role as a positive regulator of adipogenesis through modifying CAP-related insulin signaling.

## Results

### Interaction between STAP-2 and CAP

**Figure 1:**
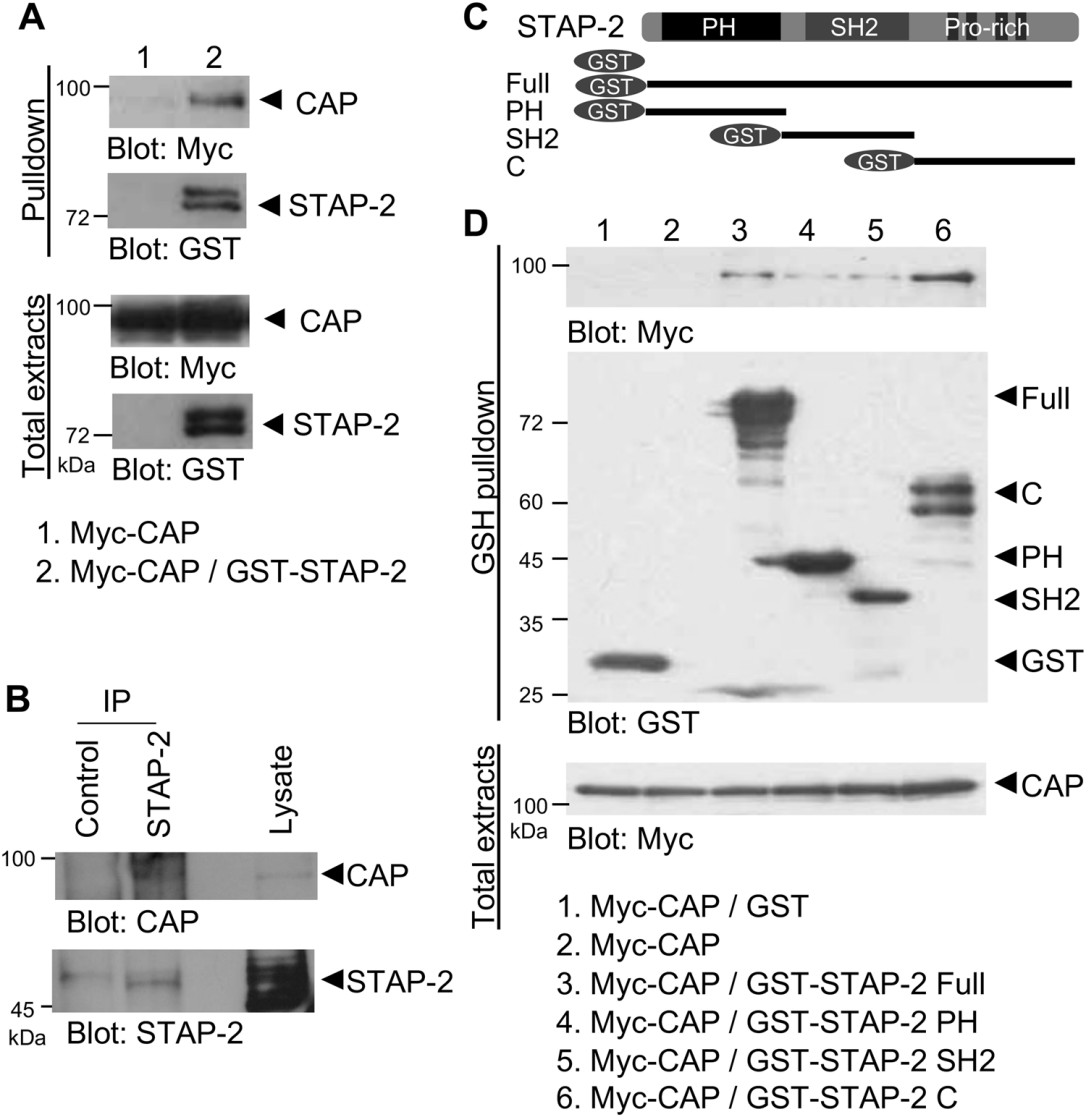
Interaction between STAP-2 and CAP (**A**) HEK293T cells were transfected with indicated plasmid. At 36 h after transfection, cells were lysed and pulled down with GSH-sepharose, and immunoblotted with anti-Myc and anti-GST antibodies. (**B**) At 6 days after adipocyte induced 3T3-L1 cells were lysed and immunoprecipitated with normal rabbit IgG or anti-STAP-2 antibody. Then, immunoprecipitants were immunoblotted with anti-CAP and anti-STAP-2 antibodies. (**C**) Schematic images of GST-fused STAP-2 deletion constructs. (**D**) The HEK293T cells were transfected with indicated plasmid. At 36 h after transfection, cells were lysed and pulled down with GSH-sepharose, and immunoblotted with anti-Myc and anti-GST antibodies.

Our yeast two-hybrid screening system has identified STAP-2-binding proteins, including CAP^9^. Thus, we focused on the CAP protein and analyzed the interaction between STAP-2 and CAP. First, we confirmed the binding of STAP-2 to CAP in mammalian cells. Myc-tagged CAP was transfected into HEK293T cells with or without GST-tagged STAP-2, and the lysates were pulled down with GSH-Sepharose (Fig. 1A). The GSH-pulled down precipitates contained both CAP and STAP-2 in STAP-2 transfected cell lysate but not in control. Next, the association between endogenous CAP and STAP-2 was assessed in lysates of mouse fibroblast 3T3-L1 cells. On day 6, after adipose differentiation, the 3T3-L1 cells were lysed and immunoprecipitated using normal rabbit IgG or anti-STAP-2 antibodies. The immunoprecipitates were resolved by SDS-PAGE and immunoblotted with anti-CAP and anti-STAP-2 antibodies (Fig. 1B). CAP proteins were co-immunoprecipitated with STAP-2 immunoprecipitants but not in control, indicating that CAP and STAP-2 interact endogenously in 3T3-L1 cells. STAP-2 is an adaptor protein that interacts with the typical domain structures. To investigate which domains in STAP-2 are indispensable for the interaction with CAP, we used STAP-2 truncated constructs fused to GST: GST-STAP-2 PH, GST-STAP-2 SH2, and GST-STAP-2 C (Fig. 1C). HEK293T cells were transfected with Myc-CAP and a series of STAP-2 deletion mutants, lysed, and pulled down using GSH-Sepharose. The interaction between CAP and STAP-2 Full was confirmed. In addition, CAP strongly bound to the C-terminal region of STAP-2 (Fig. 1D). Thus, STAP-2 interacts with CAP through its C-terminal protein-rich region.

### Ternary complex of STAP-2 with CAP and c-Cbl

**Figure 2:**
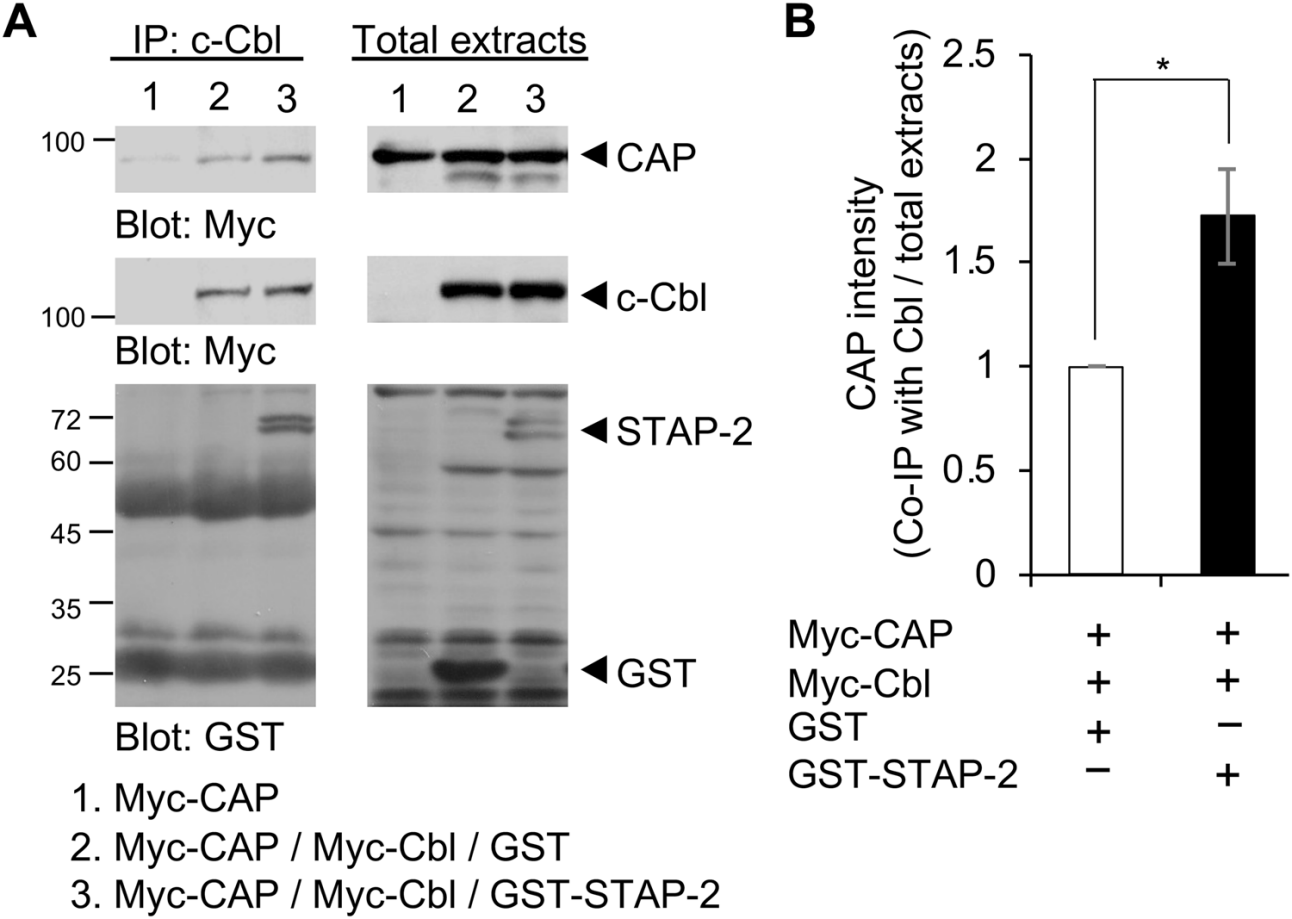
Ternary complex of STAP-2 with CAP and c-Cbl (**A**) HEK293T cells were transfected with indicated plasmid. At 36 h after transfection, cells were lysed and immunoprecipitated with anti-c-Cbl antibody, and immunoblotted with anti-Myc and anti-GST antibodies. (**B**) The graph shows the quantification of CAP protein levels in the immunoprecipitates normalized to total cell lysate. Independent experiments from 3 replicates are summarized and presented as mean ± SEM. *p < 0.05, Student’s two-tailed t test.

We previously reported that STAP-2 interacts with c-Cbl to regulate signaling pathways in mammalian cells^19,20^. Evidence that STAP-2 associates with CAP suggests that STAP-2 may make a ternary complex with CAP and c-Cbl. To test this possibility, HEK293T cells were co-transfected with Myc-Cbl and Myc-CAP, together with control GST or GST-STAP-2, and then lysed and immunoprecipitated with an anti-c-Cbl antibody (Fig. 2A). The c-Cbl protein was immunoprecipitated with an anti-c-Cbl antibody and co-immunoprecipitated with both CAP and STAP-2 in HEK293T cells. The amount of precipitated CAP was significantly higher in GST-STAP-2 transfected cells than in GST-transfected control cells (Fig. 2B). These data suggest STAP-2 increases the interactions between CAP and c-Cbl by bridging them.

### Enhancement of insulin-stimulated GLUT4 translocation by STAP-2 expression

**Figure 3:**
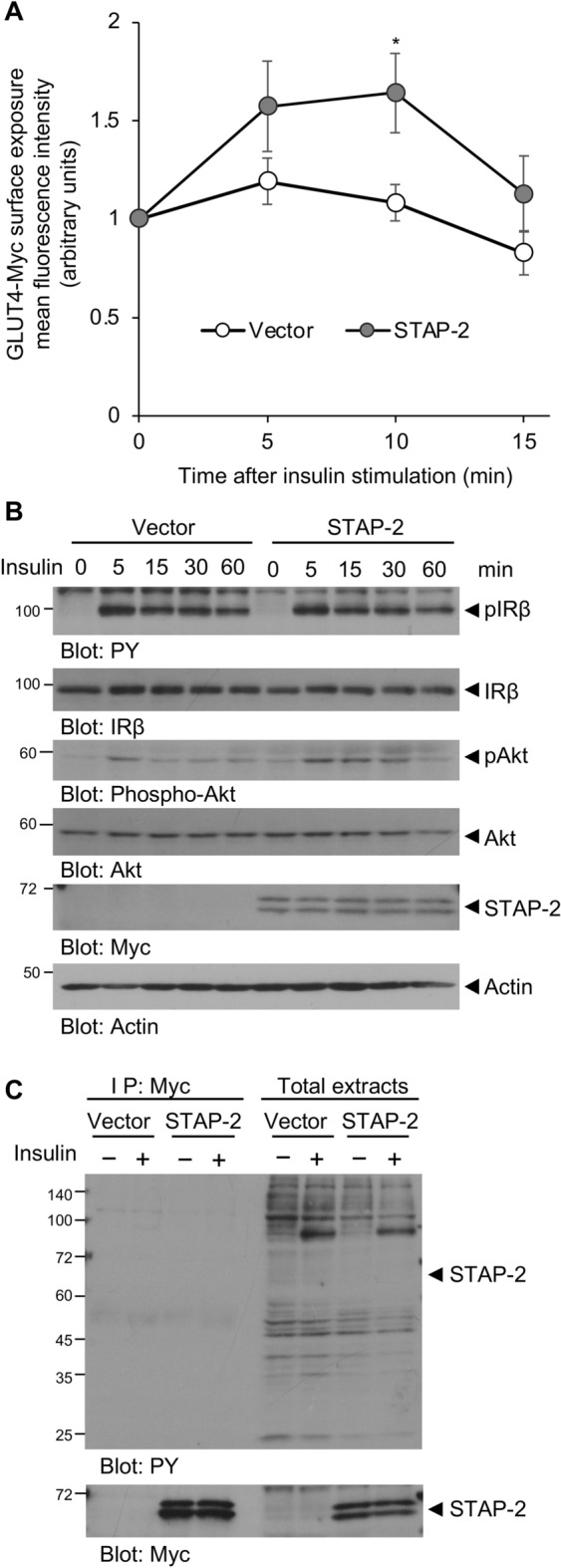
Enhancement of insulin signaling by STAP-2 expression (**A**) Hep3B cells transfected with empty vector (Vector) or stably expressing Myc-STAP-2 (STAP-2) in 6-well plates at 1 × 10^5^ cells/well were transfected with GLUT4-Myc-GFP. Thirty 6 h after transfection, cells were starved for 12 h and stimulated with insulin (1 µg/ml) for the indicated periods. Cells were fixed and stained with anti-Myc antibody, then Myc stained cells in the GFP-positive cells at each time point was evaluated using a FACSCalibur. The surface-exposed GLUT4-Myc levels of each time point were normalized to those of untreated controls. quantification of CAP protein levels in the immunoprecipitates normalized to total cell lysate. Independent experiments from 4 replicates are summarized and presented as mean ± SEM. *p < 0.05, Student’s two-tailed t test. (**B**) Hep3B/Vector or /STAP-2 cells were stimulated with insulin (300 ng/ml) for the indicated periods. Cells were lysed and immunoblotted for individual antibodies. (**C**) Hep3B/Vector or /STAP-2 cells were stimulated with insulin (300 ng/ml) for 5 min. Cells were lysed and immunoprecipitated with anti-Myc antibody, then immunoblotted with anti-PY and anti-Myc antibodies.

In response to insulin, the insulin receptor recruits CAP and c-Cbl for transducing signals. CAP/c-Cbl complex leads to the translocation of GLUT4 to the plasma membrane and induces glucose uptake^12,15,21^. Since STAP-2 interacts with both CAP and c-Cbl, STAP-2 may have a function in the insulin signaling pathway. To test this hypothesis, we evaluated the translocation of GLUT4 using the GLUT4-Myc-GFP reporter system^22^. Human hepatocytes Hep3B cells, which respond to insulin stimulation, stably overexpressing STAP-2 or control Vector were stimulated with insulin for the indicated times, then monitored the translocation of GLUT4 by flow cytometry. The translocation of GLUT4 to the plasma membrane after insulin stimulation was significantly increased in STAP-2 overexpressed cells compared to control cells (Fig. 3A and Supplementary Fig. S1). The phosphorylation levels of IRβ after insulin stimulation was analyzed, because expression of STAP-2 affected insulin-induced translocation of GLUT4. Hep3B cells were stimulated with insulin for the indicated times, lysed, and immunoblotted with anti-PY antibody. The phosphorylation of IRβ was comparable between Vector and STAP-2 throughout the time periods, while phosphorylation of Akt was increased in STAP-2-expressing cells (Fig. 3B). The tyrosine residues of STAP-2 can be phosphorylated by several kinases, and this phosphorylation is sometimes required for the functional activities of STAP-2. Thus, Hep3B/STAP-2 cells were used to monitor the tyrosine phosphorylation of STAP-2 after insulin stimulation. The cells were treated with insulin for 5 min, lysed, immunoprecipitated with an anti-Myc antibody, resolved by SDS-PAGE, and immunoblotted with an anti-PY antibody (Fig. 3C). Although phosphorylation of insulin receptor (IR) β was observed following insulin stimulation, phosphorylation of STAP-2 was not detected in the Myc-STAP-2-immunoprecipitates. Therefore, STAP-2 upregulated the insulin-mediated GLUT4 translocation without changing the phosphorylation of IRβ and STAP-2 in Hep3B cells.

### A functional role of STAP-2 in in vitro adipogenesis

**Figure 4:**
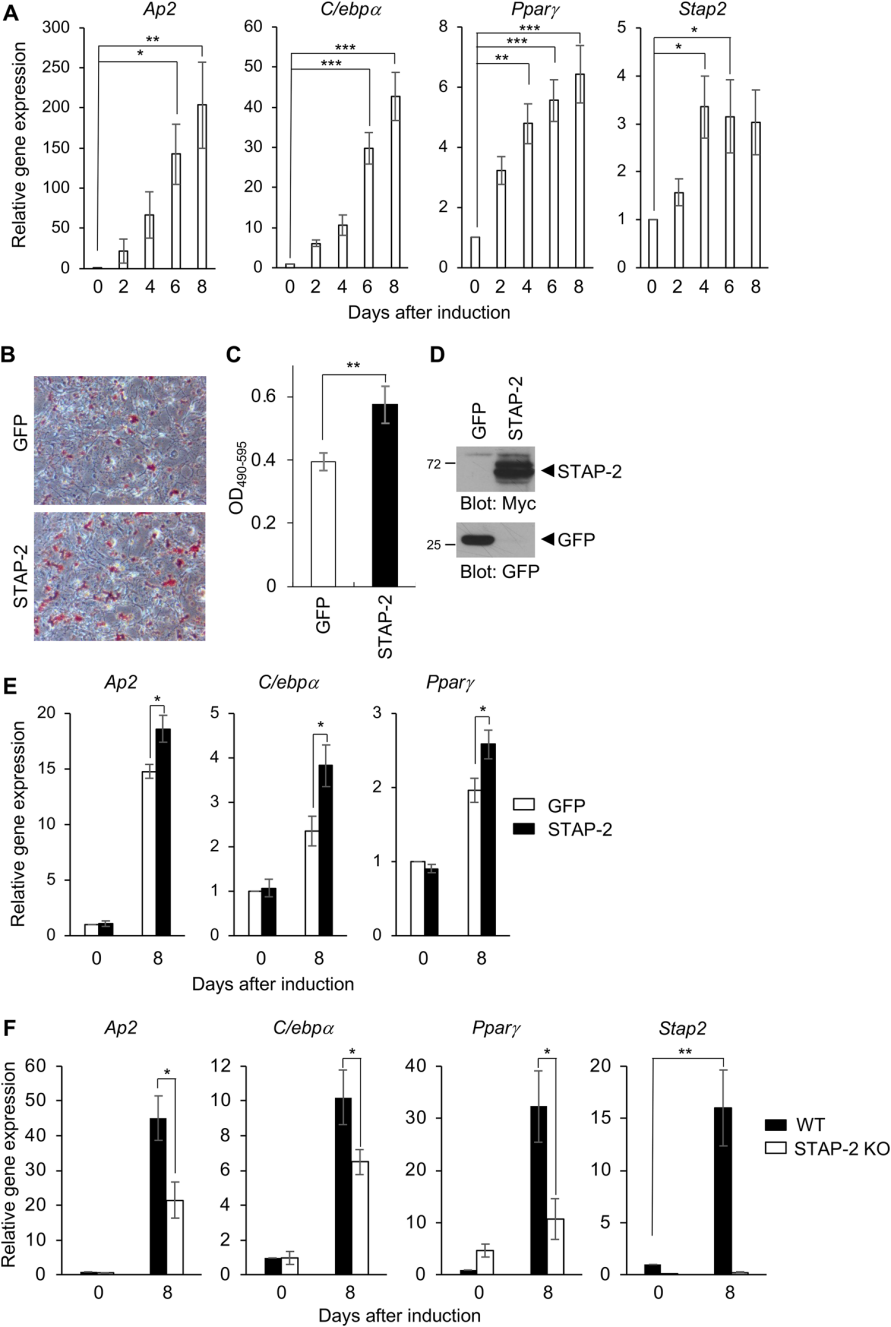
A functional role of STAP-2 in in vitro adipogenesis. (**A**) Mouse 3T3-L1 cells were induced for adipose differentiation. At 0, 2, 4, 6 and 8 days after differentiation, mRNA was extracted and analyzed the induction of *Ap2*, *C/ebpα*, *Pparγ* and *Stap2* genes. Independent experiments from 5 replicates are summarized and presented as mean ± SEM. *p < 0.05, **p < 0.01, ***p < 0.005, one-way ANOVA followed by Dunnett's test (Day 0). (**B**, **C**) Mouse 3T3-L1 cells were retrovirally transduced with GFP or Myc-STAP-2 and induced into adipocyte. At 8 days after differentiation, cells were stained with Oil Red O (**B**). The Oil Red O dye was extracted and measured at OD490-595. Data are presented as the mean ± SEM, n = 3. *p < 0.05, Student’s two-tailed t test (**C**). (**D**) Retrovirally transduced GFP and STAP-2 in 3T3-L1 cells were detected by immunoblot using anti-Myc and anti-GFP antibodies. (**E**) GFP or STAP-2 was retrovirally transduced into 3T3-L1 cells and cells were cultured for adipose differentiation. At 0 or 8 days after differentiation, mRNA was extracted and analyzed the induction of *Ap2*, *C/ebpα* and *Pparγ* genes. Independent experiments from 5 replicates are summarized and presented as mean ± SEM. *p < 0.05, Student’s two-tailed t test. (**F**) MEFs were taken from WT and STAP-2 KO mice and cultured for adipose differentiation. At 0 or 8 days after differentiation, mRNA was extracted and analyzed the induction of *Ap2*, *C/ebpα*, *Pparγ* and *Stap2* genes. Independent experiments from 3 replicates are summarized and presented as mean ± SEM. *p < 0.05, **p < 0.01, Student’s two-tailed t test.

As STAP-2 may play a functional role in insulin signaling, we assessed the effect of STAP-2 on the differentiation of adipocyte differentiation in the presence of insulin. To this end, we used mouse fibroblast 3T3-L1 cells for adipogenesis experiments. During adipose differentiation, mRNAs were collected every other day and analyzed for the induction of adipogenic and *Stap2* genes by RT-qPCR (Fig. 4A). The expression levels of adipogenic genes *Ap2*, *C/ebpα*, and *Pparγ* were increased during differentiation, and notably, *Stap2* mRNA level was also increased in 3T3-L1 cells.

Increased *Stap2* mRNA levels during adipocyte differentiation suggest that STAP-2 may play a functional role in adipogenesis. 3T3-L1 cells were retrovirally transduced with GFP or STAP-2 and then induced their differentiation into adipocytes. On day 8, after the induction of adipocyte differentiation, cells were fixed and stained with Oil Red O, and the dye was extracted to measure the OD for quantification (Fig. 4B–D). A greater number of Oil Red O-positive cells was detected in STAP-2-overexpressing 3T3-L1 cells. The effect of STAP-2 expression on the induction of adipogenic genes was examined in STAP-2-overexpressing 3T3-L1 cells. The expression levels of *Ap2*, *C/ebpα*, and *Pparγ* were significantly increased in STAP-2-overexpressing 3T3-L1 cells at day 8 after the induction of differentiation (Fig. 4E). Since ectopic overexpression of STAP-2 in 3T3-L1 cells resulted in a significant increase in adipocyte differentiation, we examined the effect of deletion of STAP-2 on adipogenesis. Mouse embryonic fibroblasts (MEFs) obtained from WT and STAP-2 knock-out (KO) mice were subjected to adipogenic experiments. Consistent with the overexpression study in 3T3-L1 cells, deletion of STAP-2 decreased mRNA expression of *Ap2*, *C/ebpα*, and *Pparγ* at day 8 after adipocyte induction (Fig. 4F). Notably, *Stap2* mRNA levels were significantly increased in WT MEFs eight days after differentiation. These data suggested that STAP-2 is a positive regulator of adipocyte differentiation.

### Function of STAP-2 in high-fat diet mouse

**Figure 5:**
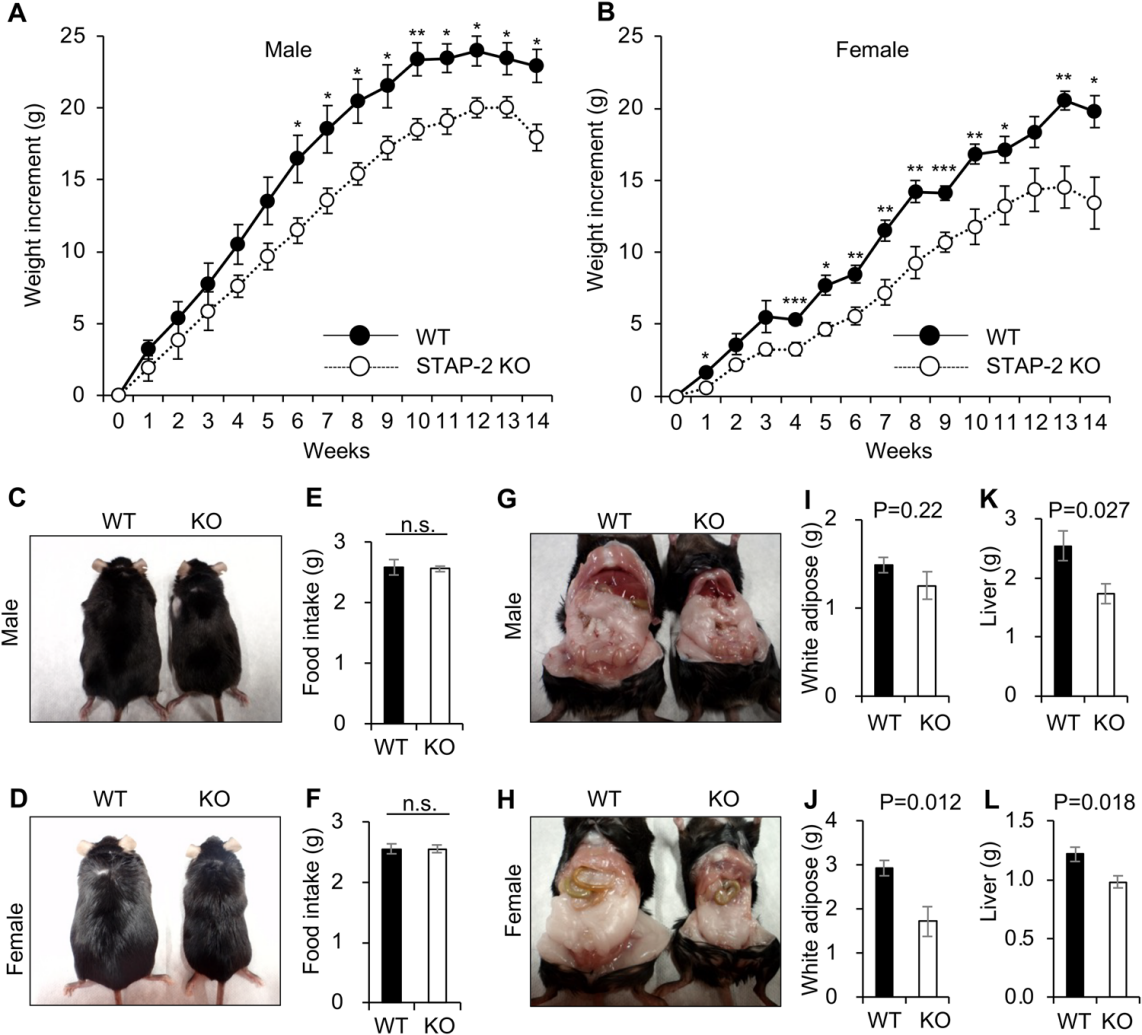
Function of STAP-2 in high fat diet (HFD) mouse. (**A**, **B**) Age matched male (**A**) and female (**B**) from WT and STAP-2 KO mice were fed high fat diet and their weight was measured every week for the indicated periods. Error bars represent SEM, n = 5. *p < 0.05, **p < 0.01, ***p < 0.005, Student’s two-tailed t test. (**C**, **D**) Representative photos of HFD fed animals at 14 weeks after HFD feeding. (**E**, **F**) After 6 weeks HFD feeding, daily food intake was monitored for 7 days. Error bars represent SEM, n = 5. (**G**, **H**) At 14 weeks after HFD feeding, animals were sacrificed and dissected liver and adipose tissues. Representative photos of HFD fed animals in male (**G**) and female (**H**). (**I**–**L**) White adipose tissues from male (**I**) and female (**J**), and livers from male (**K**) and female (**L**) were taken and weighed. Error bars represent SEM, n = 5. Student’s two-tailed t test.

Because STAP-2 plays an essential role in adipose differentiation in vitro, we examined in vivo involvement of STAP-2 in adipose tissues. WT and STAP-2 KO animals were fed a high-fat diet (HFD), and their body weights were monitored weekly for 14 weeks (Fig. 5A,B). Compared to WT mice, STAP-2 KO animals showed a much lower weight increase and body size in both males and females (Fig. 5A–D). Food intake weights were comparable among groups (Fig. 5E,F). Comparing normal diet (ND)-fed animals, the body weights of STAP-2 KO male animals were lower than WT animals at 8 weeks, but not in female animals (Supplementary Fig. S2). Fourteen weeks after HFD feeding, the animals were sacrificed, and their visceral white adipose tissues and livers were dissected and weighed (Fig. 5G–L). The weight of visceral white adipose tissue tended to be reduced in STAP2 KO mice, with significant difference in female mice (Fig. 5I,J). The significant reduce of liver weight was observed in both female and male STAP2 KO mice (Fig. 5K,L). Thus, the deletion of STAP-2 suppressed HFD-induced increases in body and liver weights. Because *Stap-2* mRNA was increased during adipocyte differentiation in vitro*, Stap2* mRNA may increase in adipose tissue of HFD feeding mice. Therefore, we evaluated *Stap2* mRNA expression in white adipose tissues (WAT) from HFD feeding mice using publicly available RNA-seq data (NCBI GEO; GSE129573)^23^. As expected, *Stap2* mRNA expression in inguinal WAT from HFD feeding for 15 weeks mice was 2 times higher than that of normal diet animals (Supplementary Fig. S3).

## Discussion

The major finding of the present study was the ability of STAP-2 to enhance the physical association between CAP and c-Cbl as a signaling complex. CAP was initially cloned as a c-Cbl-binding protein with a yeast two-hybrid screen from a 3T3-L1 cDNA expression library using c-Cbl as bait^24^. Here, we showed that the protein-rich region of STAP-2 is necessary for its interaction with CAP, whereas we previously showed that STAP-2 interacts with c-Cbl through its PH and SH2-like domains^20^. Since the proline-rich region generally recognizes SH3 domains, we supposed that the CAP SH3 domain may be an interacting motif for STAP-2. Thus, STAP-2 is likely to form a ternary complex with CAP and c-Cbl using its different domain structures: the STAP-2 proline-rich region for CAP and STAP-2 PH and SH2-like domains for c-Cbl. In the LPS/Toll-like receptor 4 (TLR4) signaling cascade, STAP-2 bridges MyD88 and IKKβ, followed by enhancing cytokine production in macrophages^25^. Similar to LPS/TLR4 signaling, our results suggest that STAP-2 functions as a linker protein to increase CAP/c-Cbl interactions and to enhance insulin-mediated adipogenesis.

c-Cbl, ubiquitously expressed in mammalian tissues, is a key cellular signaling regulator, an adaptor protein, or an E3 ubiquitin ligase. Studies have established that c-Cbl has different functions in several cellular signaling pathways, including receptor tyrosine kinases, protein tyrosine kinases, T cell receptors, B cell receptors, and integrins^26–29^. EGF receptor (EGFR) is a well-studied target of c-Cbl, which directly binds to phosphorylated EGFR and induces ubiquitin-dependent degradation. STAP-2 increases EGFR phosphorylation in response to EGF and upregulates EGFR signaling in the prostate cancer cell line DU145. STAP-2 associates with EGFR and reinforces its stability by inhibiting c-Cbl-mediated ubiquitination and degradation of EGFR^7^. In T-cells, STAP-2 interacts with c-Cbl and induces the ubiquitination and degradation of focal adhesion kinase (Fak) proteins, reducing integrin-mediated T-cell adhesion^19^. It has also been demonstrated that c-Cbl controls STAP-2 protein content through proteasomal degradation and affects STAP-2-regulating STAT3 transcriptional activation^20^. Accumulating data suggest that the STAP-2/c-Cbl axis plays critical but distinct roles in different signaling pathways. Here, we propose a novel STAP-2/c-Cbl/CAP complex that enhances insulin signaling.

The functional roles of adaptor proteins containing a PH and SH2 domain (APS) and Src homology 2-B adaptor protein (SH2-B) in insulin signaling and adipogenesis have been well studied^30–36^. Although both SH2-B and APS are tyrosine-phosphorylated by insulin, their phosphorylation levels differ^37^. Tyrosine phosphorylation of APS is strongly detected even at lower insulin doses, whereas SH2-B phosphorylation is weak. Tyrosine phosphorylation of STAP-2, induced by several kinases, is sometimes crucial for regulating STAP-2-mediating signal transductions^1,4,7,9,38^. However, tyrosine phosphorylation of STAP-2 was not induced by insulin stimulation in Hep3B cells, suggesting that STAP-2 phosphorylation may be dispensable for regulating insulin receptor signaling. Since STAP-2 KO mice are viable and fertile without any reported abnormalities under steady-state conditions, it is thought that the functions of STAP-2 emerge in some abnormal situations, such as infection, inflammation, and tumorigenesis. Notably, STAP-2 is an inducible protein^1^, and *Stap2* mRNAs are induced in 3T3-L1 cells and MEFs during differentiation into adipocytes. Thus, the functional activity of STAP-2 in insulin signaling is likely controlled by the amount of protein but not by tyrosine phosphorylation, and STAP-2 appears to be a component of a positive feedback signaling loop for regulating adipogenesis.

The binding of insulin to IRα induces the tyrosine kinase activity of IRβ followed by transphosphorylation of IRβ^39^. In response to insulin, both APS and SH2-B augment insulin signaling by enhancing the autophosphorylation of IR^37^. Insulin-induced phosphorylation levels of IRβ were comparable between vector and STAP-2 transfected Hep3B cells, suggest that STAP-2 enhances the activation of downstream signaling molecules of IRβ but not its transphosphorylation. Therefore, STAP-2 acts differently than APS and SH2-B in insulin signaling. After insulin stimulation, the insulin receptor recruits CAP and c-Cbl, then CAP/c-Cbl complex translocates to a caveolin-rich membrane compartment leading to make a ternary complex with flotillin. This signaling complex is essential for the translocation of GLUT4 to the plasma membrane and induces glucose uptake^12,15,21^. Our current studies demonstrated that STAP-2 enhanced CAP/c-Cbl interaction, and the insulin-stimulated GLUT4 translocation to the plasma membrane is augmented by STAP-2 expression. These data suggest that STAP-2 plays a role of a component of the CAP/c-Cbl-mediated insulin-stimulated glucose transport signal.

After HFD feeding, STAP-2 KO mice exhibited lower body weights than WT mice. The evidence for the positive regulation of adipogenesis in 3T3-L1 cells and MEFs by STAP-2 expression supports the results of the HFD-feeding experiment. However, we did not monitor the serum levels of glucose homeostasis parameters such as insulin, glucose, glucagon, and leptin or energy expenditure, including metabolic rate and ambulatory activity. These parameters may have affected the reduction in body weight of STAP-2 KO mice during HFD feeding. Although c-Cbl is a key molecule for regulating insulin-stimulated signaling in 3T3-L1 cells, c-Cbl deficient mice showed improvements in whole-body insulin action and higher energy expenditure^40^. Bone marrow transplantation experiments have revealed that deleting the *Cap* gene from macrophages protects against HFD-induced insulin resistance^41^. STAP-2 is expressed in hepatocytes and macrophages, where it plays a functional role. Further studies are needed to provide convincing evidence for STAP-2 KO mice in HFD-feeding experiments.

## Methods

### Reagents, antibodies and expression plasmids

Recombinant human insulin and dexamethasone (Dex), and 3-isobutyl-1-methylxanthine (IBMX) were purchased from Wako chemicals (Osaka, Japan), and Santa Cruz Biotechnology (Santa Cruz, CA, USA), respectively. Mouse anti-phosphotyrosine (PY) mAb (4G10), anti-CAP (sorbs1), and anti-STAP-2 antibodies were from Milipore (Temecula, CA), Proteintech (Rosemont, IL), and Novus biologicals (Centennial, CO), respectively. Anti-phospho-Akt (Ser473) (D9E), anti-c-Cbl and anti-Myc (9B11) antibodies were purchased from Cell Signaling Technologies (Beverly, MA, USA). Anti-CAP (G-3), anti-Akt1 (C20), anti-IRβ (11B6), anti-GFP (B-2) and anti-GST (Z-5) antibodies were obtained from Santa Cruz Biotechnology (Santa Cruz, CA, USA). Anti-Myc (9E10) and anti-β-Actin antibody was from Sigma-Aldrich (St. Louis, MO, USA). A series of GST-STAP-2 deletion mutants, Myc-Cbl and pMX-Myc-STAP-2 constructs were described previously^3,42^. Expression vectors for Myc-tagged-CAP was generated by PCR methods and sequenced (primer sequences are available upon request). GLUT4-Myc-GFP construct was kindly provided by Jonathan Bogan (Yale University).

### Cell culture, transfection and retroviral transduction

Human embryonic kidney 293 T (HEK293T and human hepatoma cell line Hep3B were maintained in DMEM containing 10% FBS, 100 U/ml penicillin and 100 μg/ml streptomycin. Mouse fibroblast 3T3-L1 obtained from JCRB cell bank and mouse embryonic fibroblast (MEF) from WT or STAP-2 knock out mice were maintained in DMEM with low glucose (1 g/l) containing 10% FBS, 100 U/ml penicillin and 100 μg/ml streptomycin. Stable Hep3B cells overexpressing Myc-STAP-2 were established as described previously^4^. Briefly, Hep3B cells were transfected with the pcDNA3 empty vector or Myc-tagged human STAP-2 by electroporation (BioRad) and selected in the above medium with G418 (0.5 mg/ml), then single-cell clones were established by a limiting dilution technique. Plasmids were transfected into HEK293T cells with polyethylenimine (PEI, Polysciences Inc). Retroviral transduction was performed as previously described^42^.

### Immunoprecipitation, immunoblotting and pull-down assay

The immunoprecipitation and Western blotting assays were performed, as described previously^43^. Briefly, cells were harvested and lysed in a lysis buffer (50 mM Tris–HCl, pH 7.4, 0.15 M NaCl, containing 1% NP-40) and centrifuged at 20,000×*g* for 20 min at 4 °C. For immunoprecipitation, antibody and protein G-sepharose mixture was added to lysates and incubated for 2 h at 4 °C with gentle rotation. For GST pull-down assay, GSH-sepharose was added to lysates and incubated for 2 h at 4 °C with gentle rotation.

The beads were washed three times and the complexes were then resolved by SDS-PAGE. After transfer, the PVDF membranes (PerkinElmer; Boston, MA) were incubated in the 5% skimmed milk or 3% BSA blocking buffer 30 min at RT and immunoblotted with the appropriate primary antibodies. Following primary antibody incubation, secondary antibodies were applied for 1 h at RT and immunoreactive proteins were visualized using an enhanced chemiluminescence detection system (Cytiva; Marlborough, MA).

### GLUT4 translocation assay

GLUT4-Myc-GFP expression plasmid was transfected in Hep3B/Vector or Hep3B/STAP-2 cells using PEI. At 36 h after transfection, cells were starved for 12 h and then stimulated with insulin (1 µg/ml) for the indicated periods. Cells were fixed with 4% paraformaldehyde and stained with anti-Myc antibody for 2 h at 4 °C. Following primary antibody incubation, Alexa594-conjugated anti-mouse IgG secondary antibody was applied for 1 h at room temperature. The mean fluorescein intensity of Myc stained cells in the GFP-positive cells at each time point was evaluated using a FACSCalibur cytometer (BD Biosciences) and analyzed with Cell Quest software. The Myc-expression levels of each time point were normalized to those of untreated controls.

### Adipocyte differentiation

MEF or 3T3-L1 cells were induced into adipocyte 2 days after confluence. Cells were cultured in low glucose DMEM with 10% FBS, 0.5 mM IBMX, 0.1 mM Dex, 10 µg/ml insulin for 2 days, then the medium was changed with low glucose DMEM with 10%FBS, 10 µg/ml insulin for 2 days. Thereafter, the medium was changed with low glucose DMEM with 10%FBS every 2 days.

### Oil red O staining

Differentiated 3T3-L1 adipocytes were fixed with 4% paraformaldehyde and stained with Oil red O solution (2 mg/ml). Quantification of oil red o staining was performed as described previously^44^. Briefly, to determine the level of staining, the stained dye was extracted from adipocytes by adding isopropanol. The absorbance of the extract was measured at 490–595 nm using a microplate reader.

### RT-PCR and quantitative PCR

Total RNA was prepared according to the TRI Reagent protocol (Takara; Japan) and subjected reverse transcriptase (RT)-PCR using the ReverTra Ace (Toyobo; Japan). cDNA of each sample was then used for real-time qPCR with THUNDERBIRD Next SYBR qPCR Mix (Toyobo) on a Bio-Rad CFX Connect Real-Time PCR Detection System using standard cycles. Each sample was loaded in duplicates. The primers used for RT-PCR were as follows: mouse *Ap2*-5′, TGAAATCACCGCAGACGACA; mouse *Ap2*-3′, CTCTTGTGGAAGTCACGCCT; mouse *C/ebpα*-5’, GAGGGGAGGGACTTAGGTGT; mouse *C/ebpα*-3′, TGCCCCCATTCTCCATGAAC; mouse *Pparγ*-5′, GACGCGGAAGAAGAGACCTG; mouse *Pparγ*-3′, GTGTGACTTCTCCTCAGCCC; mouse *Stap2*-5′, GTTGCCTCAACTACCTCCCC; mouse *Stap2*-3′, CTTGGGCTTCAGAGGGACTG; *Gapdh*-5′, GAAATCCCATCACCATCACCATCTTCCAGG; *Gapdh*-3′, CAGTAGAGGCAGGGATGATGTTC.

### Mice and high fat diet

The generation of STAP-2-deficient mice was described previously^1^. All animal experiments were approved by the Institutional Animal Care and Use Committee of Hokkaido University (Approval No. 18-0024). STAP-2-deficient mice were housed and bred in the Pharmaceutical Sciences Animal Center of Hokkaido University. All animals were maintained under pathogen-free conditions and in compliance with national and institutional guidelines. All protocols were approved by the Hokkaido University animal ethics committee and performed in accordance with relevant guidelines and regulations and ARRIVE guidelines. For high fat diet study (HFD), Age matched 4 weeks old animals were fed HFD containing 60% fat by weight (HFD32, CLEA-Japan) for 14 weeks. Then, mice were euthanized with gradually increasing concentrations of isoflurane and dissected to take white adipose tissues and livers.

### Statistical analysis

Statistical comparisons included one-way ANOVA and Student’s *t* test as specified in the Figure legends using Excel or Prism software. Statistical significance was set at *P* < 0.05. All data are mean ± SEM. No statistical methods were used to calculate sample size estimates.

### Supplementary Information

Supplementary Figures.

Supplementary Information 1.

## Data Availability

All data are contained within the manuscript.
