# Supplementary material for: STAP-2 facilitates insulin signaling through binding to CAP/c-Cbl and regulates adipocyte differentiation
Source: Sci Rep. 2024 Mar 9;14:5799. doi: 10.1038/s41598-024-56533-0 (PMC10925025; doi:10.1038/s41598-024-56533-0)
Supplement: Supplementary file 1 — Supplementary Figures. [file 41598_2024_56533_MOESM1_ESM.pdf]

STAP-2 facilitates insulin signaling through binding to CAP/c-Cbl and regulates adipocyte differentiation.

Yuichi Sekine[1,*], Kazuna Kikkawa[1], Sachie Honda[1], Yuto Sasaki[2], Shoya Kawahara[2], Akihiro Mizushima[2], Sumihito Togi[3], Masahiro Fujimuro[1], Kenji Oritani[4], Tadashi Matsuda[2,*]

[1]Department of Cell Biology, Kyoto Pharmaceutical University, Kyoto, 607-8412, Japan
[2]Department of Immunology, Graduate School of Pharmaceutical Sciences, Hokkaido University, Sapporo, 060-0812, Japan
[3]Division of Genomic Medicine, Department of Advanced Medicine, Medical Research Institute, Kanazawa Medical University, Kahoku, Ishikawa, 920-0293, Japan
[4]Department of Hematology, International University of Health and Welfare, Narita, Chiba, 286-8686, Japan

*Author to whom correspondence should be addressed,
Dr. Yuichi Sekine: sekine@mb.kyoto-phu.ac.jp,
Dr. Tadashi Matsuda: tmatsuda@pharm.hokudai.ac.jp

Supplementary Figure S1

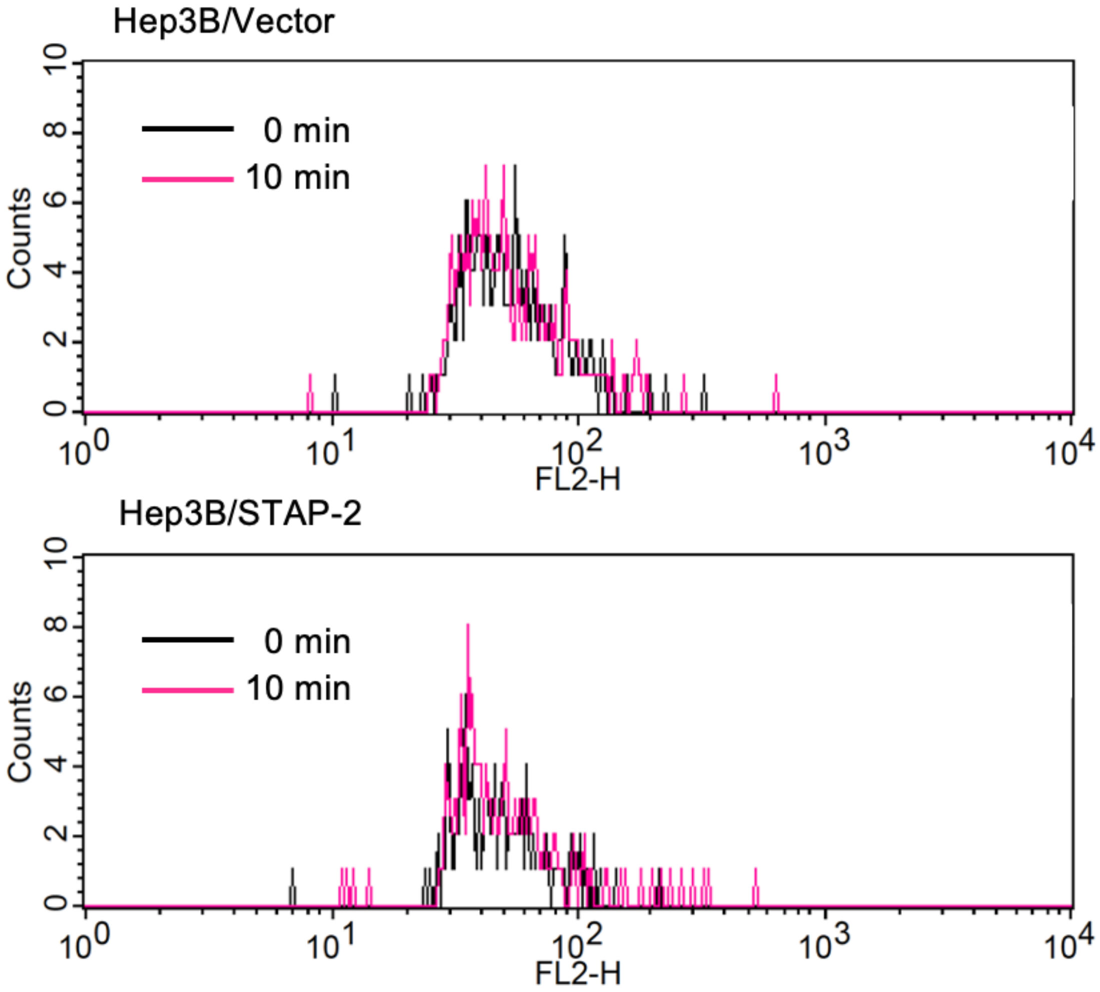

Supplementary Figure S1.

Hep3B/Vector or stably expressing STAP-2 were transfected with GLUT4-Myc-GFP. Thirty-six h after transfection, cells were starved for 12 h and stimulated without (blue line) or with insulin (1 μg/ml) for 10 min (black line). The histograms show the surface-exposed GLUT4-Myc levels in the GFP-positive cells.

Supplementary Figure S2

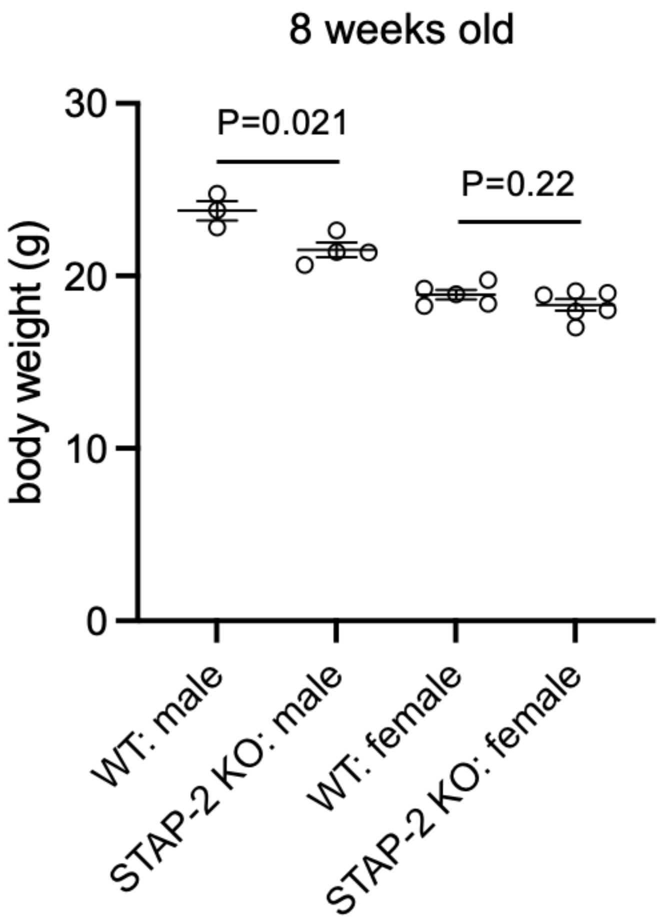

Supplementary Figure S2.

The graph shows body weights of Normal diet-fed animals at 8 weeks old with mean ± SEM. WT male (n=3) and female (n=5), STAP-2 KO male (n=4) and female (n=6). Student's two-tailed t test.

Supplementary Figure S3

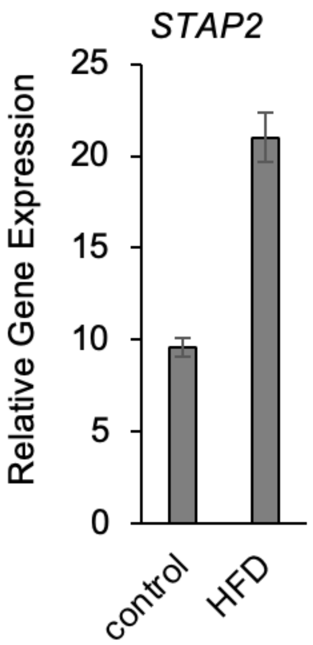

Supplementary Figure S3.

The graph shows *Stap2* mRNA expression in white adipose tissues (WAT) from HFD feeding mice using publicly available RNA-seq data (NCBI GEO; GSE129573). Error bars represent ±SEM, n=2.
