# Supplementary material for: STAP-2 facilitates insulin signaling through binding to CAP/c-Cbl and regulates adipocyte differentiation
Source: Sci Rep. 2024 Mar 9;14:5799. doi: 10.1038/s41598-024-56533-0 (PMC10925025; doi:10.1038/s41598-024-56533-0)
Supplement: Supplementary file 2 — Supplementary Information 1. [file 41598_2024_56533_MOESM2_ESM.pdf]

Figure 1

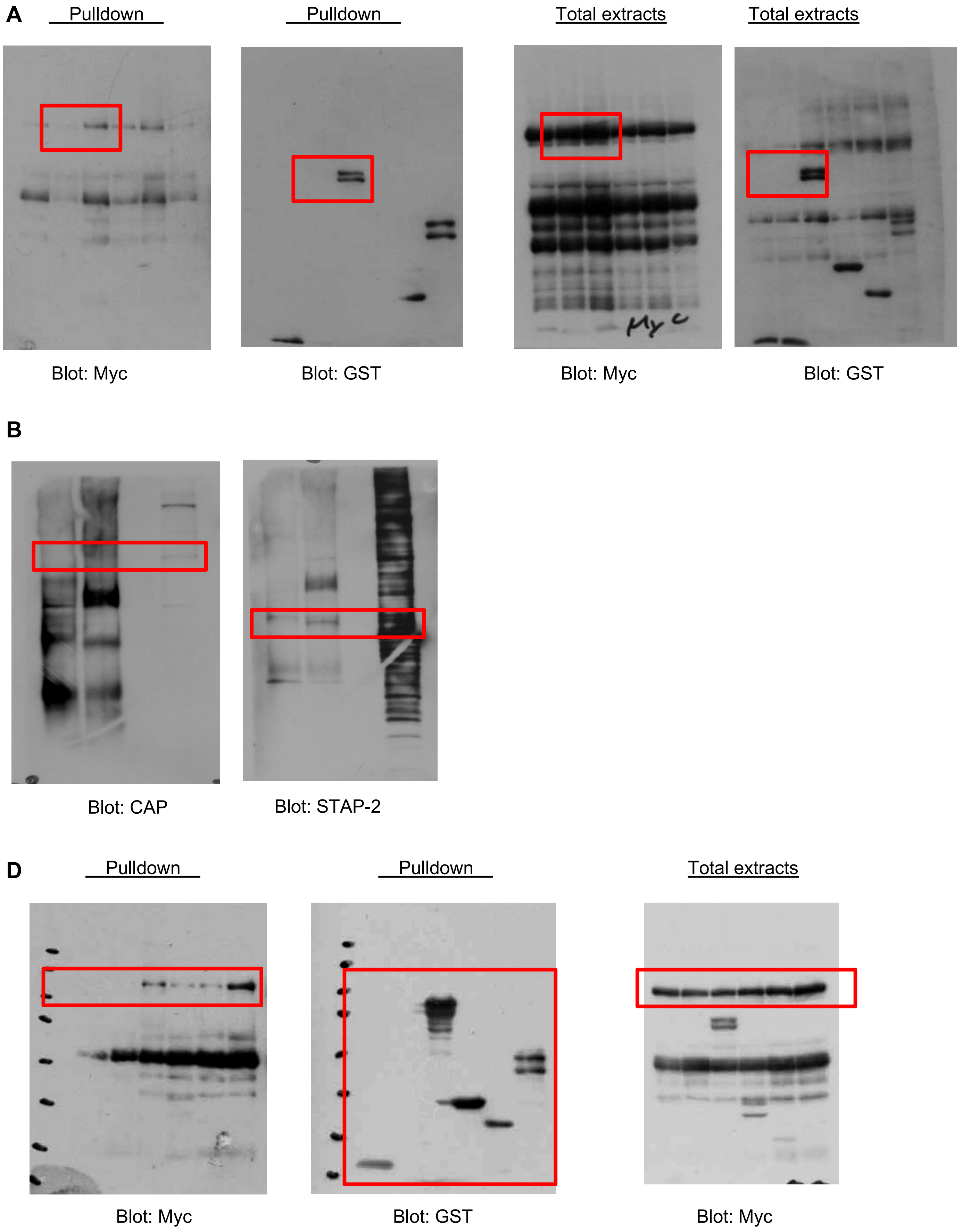
A
Pulldown
Pulldown
Total extracts
Total extracts
Blot: Myc
Blot: GST
Blot: Myc
Blot: GST
B
Blot: CAP
Blot: STAP-2
D
Pulldown
Pulldown
Total extracts
Blot: Myc
Blot: GST
Blot: Myc

Figure 2

A

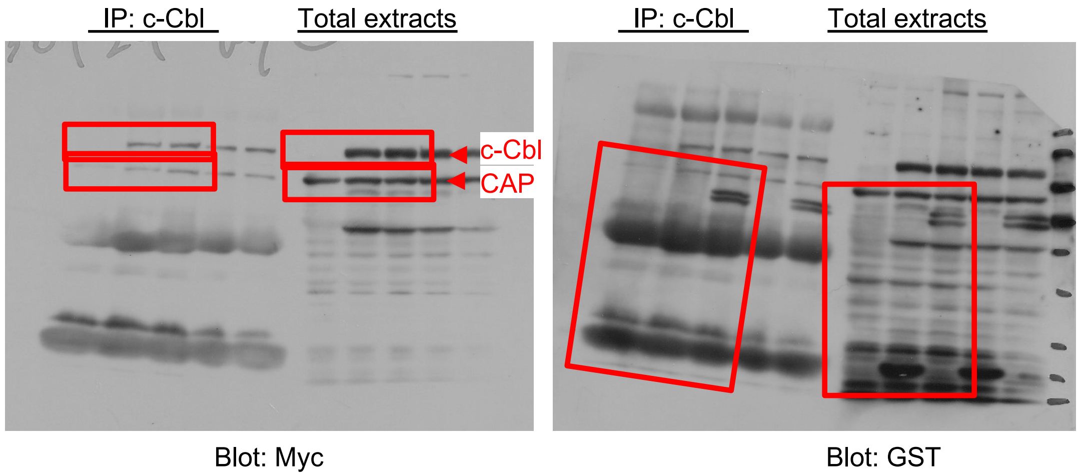

IP: c-Cbl
Total extracts
c-Cbl
CAP
Blot: Myc
IP: c-Cbl
Total extracts
Blot: GST

Figure 3

B

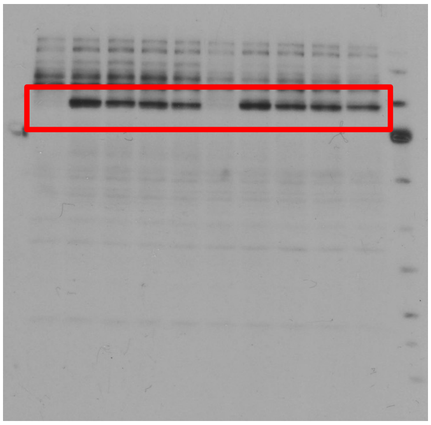

Blot: PY

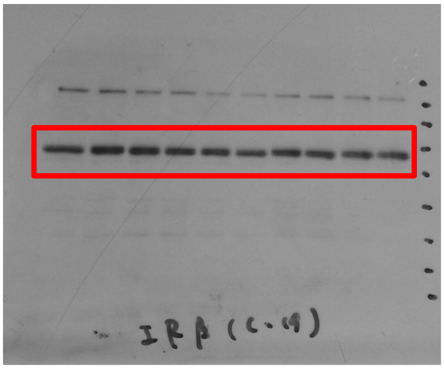

Blot: IRβ

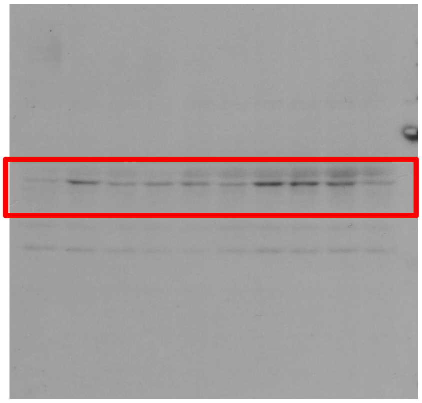

Blot: Phospho-Akt

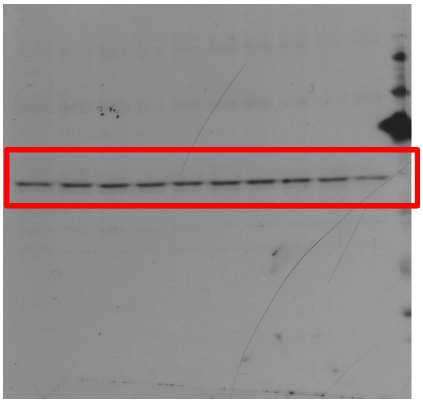

Blot: Akt

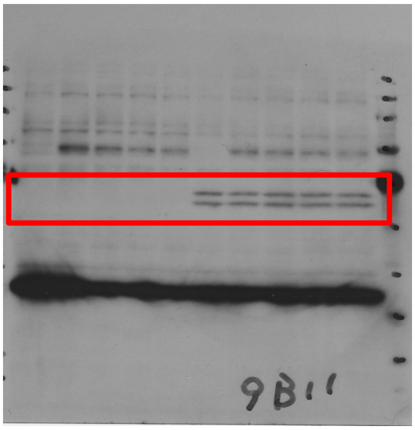

Blot: Myc

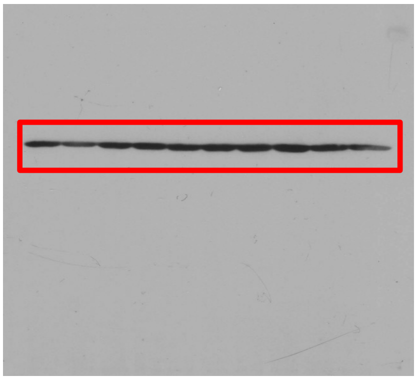

Blot: Actin

C

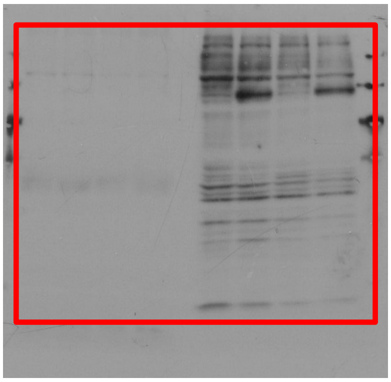

Blot: PY

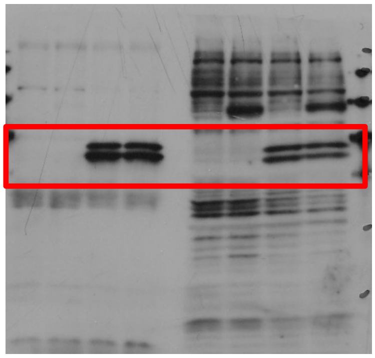

Blot: Myc

Figure 4

D

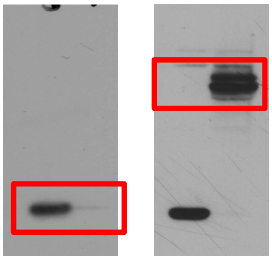

Blot: GFP Blot: Myc
